# Practical Departments

**Published:** 1905-08-26

**Authors:** 


### PRACTICAL DEPARTMENTS.

### A STRONG AND SIMPLE ADJUSTABLE LOUVRE VENTILATOR.

Mr. W. E. Peck, of 8 Broadway Avenue, London, E.C., is introducing a form of louvre ventilator, which he is importing from America, and that is worthy of attention. It is designed and constructed with all the American skill in these matters. The scarcity of labour in the United States for so many years has developed a wonderful skill, not only in designing machinery to perform work that is usually accomplished by hand, but also in economising material, without detracting in a great many cases from the utility of the apparatus constructed. In the present instance the ventilator consists of a grating with wide openings, formed in one light casing, a space being left in the front for the handle working the louvres to move. The casting is well covered with one of those black enamel varnishes, in which our American cousins are also such adepts, and it screws on to a rectangular frame, by four metal thread-screws, at the corners. The rectangular frame is fixed in the wall, and consists of two substantial end-pieces, joined by two lighter pieces, fixed longitudinally, all of iron. The heavier end-pieces support the louvres on pivots, and they are moved into any position by a brass handle at one side, working a junction-piece between the two louvres, the junction-piece moving vertically in a space provided for it. The construction of the louvres also shows American design. Each is stamped out of a piece of sheet iron, a lug for connection with the junction-piece, and two pivot pie-pieces being formed in the process of stamping.

### HOSPITAL FURNITURE AND REQUISITES.

A very useful catalogue has been published by the Hospitals and General Contracts Company, of 33 Mortimer Street, W. It is profusely illustrated, and demonstrates that the company is prepared to supply all that institutions require by way of furniture and appliances of good quality and at a reasonable price.

